# Effects of AUC-Based Vancomycin Therapeutic Drug Monitoring on AKI Incidence and Drug Utilization: A Propensity Score-Weighted Analysis

**DOI:** 10.3390/jcm14061863

**Published:** 2025-03-10

**Authors:** Hye Young Park, Bo Young Kim, Joon Young Song, Kyung Hee Seo, So Hyun Lee, Seeun Choi, Kiyon Rhew

**Affiliations:** 1 Department of Pharmacy, Korea University Guro Hospital, Seoul 08308, Republic of Korea; kby@kumc.or.kr (B.Y.K.); westgirl76@kumc.or.kr (K.H.S.); pharm23@kumc.or.kr (S.H.L.); 2 Division of Infectious Disease, Department of Internal Medicine, Korea University Guro Hospital, Korea University College of Medicine, Seoul 08308, Republic of Korea; infection@korea.ac.kr; 3 College of Pharmacy, Dongduk Women’s University, Seoul 02748, Republic of Korea; 20244093@dongduk.ac.kr

**Keywords:** acute kidney injury, vancomycin, therapeutic drug monitoring, under the curve, safety

## Abstract

**Background**: Vancomycin therapeutic drug monitoring (TDM) has traditionally relied on trough concentrations; however, recent guidelines recommend area under the curve (AUC)-based monitoring due to its potential to improve efficacy and safety. Limited studies have evaluated the impact of AUC-based dosing on clinical outcomes, particularly in South Korea. **Methods**: This single-center retrospective cohort study compared the incidence of acute kidney injury (AKI) and total vancomycin usage between patients receiving TDM based on AUC versus trough concentrations. Propensity score matching was applied to balance baseline characteristics, including age, sex, body weight, renal function, and concomitant nephrotoxic medication use. The study analyzed data from adult patients with normal renal function treated between 2021 and 2023. **Results**: After propensity score matching, AKI incidence was significantly lower in the AUC-based group (1.20%) compared to the trough-based group (5.08%) (odds ratio 0.23, 95% CI: 0.09–0.59, *p* = 0.0021). Although no significant differences were observed in treatment duration or dose adjustments, the total administered vancomycin dose was significantly reduced in the AUC-based group. This reduction likely contributed to lower AKI rates and decreased unnecessary drug exposure. **Conclusions**: Compared to trough-based dosing, AUC-based vancomycin dosing significantly reduced AKI incidence and total drug usage in adult patients with normal renal function. These findings underscore the importance of adopting AUC-based TDM in clinical practice to enhance patient safety and optimize vancomycin therapy. Further studies are needed to evaluate the broader implementation of AUC-based monitoring in diverse clinical settings.

## 1. Introduction

Vancomycin is commonly used to treat Methicillin-resistant *Staphylococcus aureus* (MRSA) infections and is known for causing nephrotoxicity, one of its major adverse effects [1,2,3]. Previous studies have reported that approximately 10% of patients receiving vancomycin experience acute kidney injury (AKI) [4], and the risk of AKI increases when vancomycin is administered alongside other nephrotoxic agents, such as aminoglycoside antibiotics or intravenous contrast agents [5]. Moreover, vancomycin-induced nephrotoxicity correlates with the dosage and duration of vancomycin treatment, and it typically occurs within the first 4 to 5 days of therapy [6].

In 2009, guidelines issued by the American Society of Health-System Pharmacists (ASHP), the Infectious Diseases Society of America (IDSA), and the Society of Infectious Diseases Pharmacists (SIDP) recommended that to achieve adequate efficacy of vancomycin against MRSA with a minimum inhibitory concentration (MIC) of 1 mg/L or less, the AUC/MIC (area under the concentration-time curve/minimum inhibitory concentration) ratio should be maintained at 400 mg×h/L or higher. Additionally, to maintain an AUC/MIC of 400 mg×h/L or above, they suggested that the trough concentration should be kept between 15 and 20 mg/L [7]. However, due to the variability in pharmacokinetic characteristics among patients receiving vancomycin and significant inter-patient variability, setting dosing strategies based solely on trough concentrations has been deemed insufficient for preventing nephrotoxicity. Studies have shown that targeting a vancomycin trough concentration of 15–20 mg/L may increase the risk of AKI due to unnecessary drug exposure [8,9].

Based on these research findings, the vancomycin TDM (therapeutic drug monitoring) guidelines published in 2020 recommended using AUC as an indicator to determine vancomycin dosage to reduce the risk of AKI, establishing a therapeutic range of 400 < AUC_24_ < 600 mg×h/L [10]. A meta-analysis published in 2015 compared the incidence of AKI between AUC-based and trough-based dosing of vancomycin [11]. This study demonstrated that determining vancomycin regimens based on the AUC/MIC ratio, rather than trough concentrations, was superior in reducing treatment failure, persistent bacteremia, and mortality, thereby improving treatment outcomes. In other words, using AUC-based dosing for vancomycin provides positive results in terms of both drug efficacy and safety.

However, determining dosage based on AUC is relatively challenging compared to using trough levels, as obtaining an AUC graph is more complex and prone to calculation errors. Additionally, multiple blood samples are required from the patient, and advanced software is necessary for dose determination, which presents significant limitations [12]. Consequently, in most clinical settings, trough levels are still predominantly used to guide vancomycin dosing. Additionally, many healthcare professionals, including pharmacists and physicians, lack sufficient awareness of the importance of AUC-based monitoring and lack plans to adopt AUC-based TDM for vancomycin [13,14,15,16].

Previous studies have shown a correlation between AUC and trough concentrations, suggesting that target trough levels, which may vary depending on dosing intervals and the elimination rate constant, can serve as a surrogate for AUC (with a target trough concentration of 10–15 mg/L) [12]. However, other studies have reported that AUC calculated using the Bayesian method, based on blood concentrations measured between 48–72 h and 72–96 h, significantly differs from AUC estimated in a steady state using a first-order pharmacokinetic method [17]. This study indicated that pharmacokinetic metrics in patients can be calculated using various methods, and AUC values predicted by different methods could potentially lead to dosing adjustment failures in approximately 50% of patients [17].

In Korea, if the dose and administration of vancomycin were set and administered based on AUC rather than trough-based, safer drug use, including a reduction in the incidence of AKI, would be possible for patients. Despite the announcement of the 2020 vancomycin TDM guidelines, only trough calculation software was used, and vancomycin dosage was mainly determined based on the trough concentration in Korea. At this hospital, a tertiary medical institution in Korea, the pharmacy department also used Capsil^®^ to guide clinical pharmacokinetics consultations for vancomycin based on trough levels until 2022. Since then, they have acquired AUC_24_ calculation software, which they now use to guide vancomycin dosing regimens. Consequently, no study in Korea has compared the risk of AKI between AUC-based and trough-based vancomycin dosing strategies in Korean patients. This study aims to compare the incidence of AKI with these two dosing approaches to contribute to safer drug use by examining changes in dosing criteria for vancomycin.

## 2. Materials and Methods

### 2.1. Study Subjects

This study was conducted at a tertiary hospital in South Korea and included patients meeting the following criteria: (1) adult patients aged 19 years or older, (2) those who received vancomycin for at least three days, and (3) patients who received pharmacokinetic consultation from the hospital pharmacy between March 2021 and May 2023. Patients with vancomycin dosing intervals of 48 h or more and those undergoing hemodialysis, peritoneal dialysis, or continuous renal replacement therapy were excluded. Since this medical institution began AUC-based vancomycin TDM services in March 2022, patients who received TDM consultation during the transition period from trough-based to AUC-based guidance between March and May 2022 were also excluded.

Patients in this study were classified into those who received trough-based TDM consultation and those who received AUC-based TDM consultation. For trough vancomycin dosing recommendations, the target trough level was set at 15–20 mg/L using the Capsil^®^ program, with trough concentration defined as the serum level measured within 1 h before the next vancomycin dose. AUC-based vancomycin dosing was determined using Bayesian software (Precise PK^®^), allowing for a single blood draw, as with trough-based dosing plans, to set the dosage. The target AUC/MIC was set at approximately 450 mg×h/L. However, the vancomycin dosing regimen was designed to achieve an AUC/MIC of over 500 mg×h/L for severe infections such as endocarditis, pneumonia, osteomyelitis, and meningitis. TDM was typically performed twice weekly, with intervals of approximately 3–4 days. The study was conducted in accordance with the Declaration of Helsinki and was approved by the Institutional Review Board of Korea University Guro Hospital (approval number: 2024GR0183, approval date: 18 May 2024).

### 2.2. Definition of Disease and Study Outcome

This study defined the primary outcome as AKI. The diagnosis of AKI was based on the KDIGO guidelines, which include the following criteria: an increase in serum creatinine (SCr) of ≥0.3 mg/dL within 48 h or an increase in SCr to ≥1.5 times the baseline within 7 days, or a urine output of <0.5 mL/kg/hour for 6 h [18]. Comorbidities and indications for vancomycin were defined as having the disease when a doctor’s diagnosis was confirmed in the hospital records. The secondary outcomes included the total dose of vancomycin administered, the duration of vancomycin therapy, and the number of modifications to the vancomycin regimen, such as dose or dosing interval adjustments.

### 2.3. Statistical Analysis

In this study, we compared demographic and clinical characteristics distributions between the two groups (trough-based TDM group and AUC-based TDM group) and calculated the incidence of AKI. Propensity scores based on patient characteristics (age, body weight, sex, baseline SCr, concomitant use of medication increasing AKI risk, indication for vancomycin, and comorbidity) were calculated using inverse probability of treatment weighting (IPTW) and applied in binary logistic regression. Medications that increase the risk of kidney damage were divided into two risk groups. High-risk medication included aminoglycosides (gentamicin, tobramycin, amikacin, streptomycin, kanamycin), antifungals (amphotericin B), cisplatin, and contrast media. Low-risk medication included NSAIDs and diuretics. For continuous variables, the Student’s *t*-test was used, and for categorical variables, the chi-square test was applied to analyze the differences between the two groups. A Cox regression model was used to estimate the odds ratio (OR) for AKI comparing two groups and their corresponding 95% confidence intervals (CIs) adjusted by IPTW. All statistical analyses were performed using SAS^®^ 9.4 (SAS Institute Inc., Cary, NC, USA). Results were deemed statistically significant if the *p*-value was less than 0.05. Data were analyzed following the recommendations outlined in the STROBE checklist to ensure comprehensive reporting of observational data.

## 3. Results

### 3.1. Subject Characteristics

**Figure 1:**
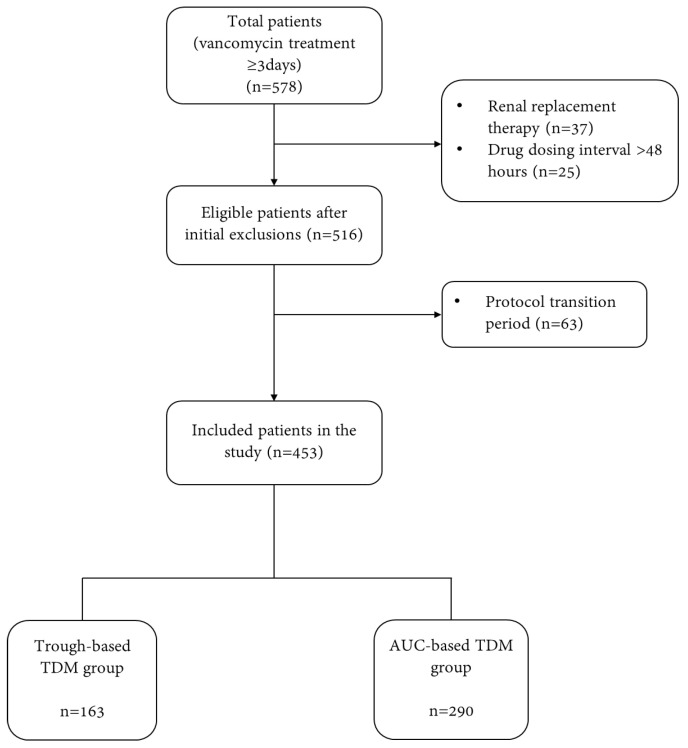
Flow diagram for study subject inclusion.

From March 2021 to May 2023, 578 adult patients received vancomycin for over three days. Among these, 125 patients were excluded, including 37 patients undergoing dialysis, 25 patients with vancomycin dosing intervals exceeding 48 h, and 63 patients who received vancomycin during the transition period for dosing protocol changes. Consequently, 453 patients were included in this study. In this study, vancomycin dosing was based on trough levels for 163 patients and AUC for 290 patients (Figure 1).

The mean age of the patients was approximately 62–63 years, with males outnumbering females by a ratio of about 2:1. The mean SCr levels ranged from 0.7 to 0.8 mg/dL, which were within the normal range. The use of concomitant medications that increase the risk of kidney injury was more frequent in the group whose dosing was based on trough levels compared to the AUC-based group. We adjusted for these patient characteristics using propensity score weighting to make the baseline characteristics of the two groups comparable. After applying propensity score weights to all patient characteristics, there were no statistically significant differences between the two groups (*p* > 0.05), and the variance ratio fell within the acceptable range of 0.5 to 2.0. In addition, the baseline characteristics of the two groups, including indications for vancomycin use, renal function markers other than SCr (eGFR, BUN, CLcr), and the distribution of comorbidities (hypertension, diabetes mellitus), were not statistically different (Table 1).

### 3.2. Incidence of Acute Kidney Injury

Before adjusting for propensity score, AKI occurred in 9 patients (5.52%) in the trough-based vancomycin dosing group (n = 163), while in the AUC-based dosing group (n = 290), three patients (1.03%) developed AKI. After adjusting for baseline characteristics using propensity score weighting, the incidence of AKI was analyzed in the groups that determined vancomycin dosing based on trough levels and AUC, respectively. In the group dosed based on trough levels, 23 cases of AKI occurred out of 455 patients, while in the AUC-based dosing group, 5 cases (1.20%) of AKI were observed. The occurrence of AKI was reduced by 77%, with an OR of 0.23 (95% CI: 0.09–0.59, *p* = 0.0021) (Table 2).

### 3.3. Secondary Outcomes

Before weighting, the total dose of vancomycin administered was significantly higher in the Trough-based TDM group (29,092 ± 33,512.8 mg) compared to the AUC-based TDM group (21,093 ± 25,478.3 mg), with a *p*-value of 0.0086. After propensity score weighting, the total dose of vancomycin administered remained significantly higher in the Trough-based TDM group (29,530 ± 54,671.7 mg) compared to the AUC-based TDM group (20,987 ± 33,124.1 mg), with a *p*-value of 0.0047. However, there was no significant difference between the two groups in terms of the total duration of vancomycin treatment or the number of modifications to the vancomycin regimen before and after propensity weighting (Table 3).

## 4. Discussion

This study showed that in adults with normal renal function, approximately 5.52% of patients who received vancomycin through trough-based TDM developed AKI. In contrast, only about 1.03% of those who received vancomycin through AUC-based TDM developed AKI. Even after adjusting for patients’ clinical characteristics using propensity score matching, AUC-based dosing significantly reduced the incidence of AKI by 23% compared to trough-based dosing in adults with normal renal function. While AUC-guided dosing did not result in statistically significant differences in the duration of vancomycin treatment or the number of dose adjustments, it was associated with a significant reduction in the total administered dose of vancomycin.

However, in clinical practice, implementing such programs faces various practical challenges, and there is little awareness of the issues associated with trough-based vancomycin dosing. As a result, trough-based dosing remains more commonly implemented. To the best of our knowledge, this study is the first in South Korea to quantitatively analyze the extent to which AUC-based dosing reduces AKI incidence in adult patients with normal renal function. The findings of this study could demonstrate that AUC-based vancomycin dosing provides significant benefits even in adults with normal renal function, challenging the perception among clinicians and pharmacists that it is not essential.

Previous studies have reported that the incidence of AKI following vancomycin administration ranges from 5% to 43% [19]. Additionally, a separate meta-analysis indicated that vancomycin administration increased the risk of AKI by 2.45 times compared to non-glycopeptide antibiotics [20]. After adjusting for confounders using propensity score matching in this study, the AKI incidence in patients receiving trough-based dosing was approximately 5.08%, compared to 1.20% in those receiving AUC-based dosing. While these absolute AKI incidence rates are lower than those reported in previous studies, this discrepancy may be attributed to the fact that all patients in this study had normal baseline renal function and that the hospital maintained continuous renal function monitoring during vancomycin therapy.

At the hospital where this study was conducted, vancomycin is classified as a restricted antimicrobial agent requiring approval from the infectious disease department. However, vancomycin can be used without approval during weekends and public holidays for up to 48–72 h. In such cases, vancomycin therapy can commence immediately for patients in need. Following AUC-based TDM implementation, clinicians were advised to administer a loading dose and request TDM within 24–48 h. Nonetheless, in many cases, blood sampling is performed after four doses at a steady state, similar to the practice when using trough-based monitoring. This clinical practice may partly explain the lack of statistically significant differences in treatment duration between the two groups. Additionally, since the duration of vancomycin administration was determined according to clinical guidelines for each treatment indication, it is expected that there was no significant difference in the administration duration between the two groups.

Despite the similarity in treatment duration, the total administered dose of vancomycin was significantly lower in the AUC-based dosing group. This reduction likely reflects decreased unnecessary drug exposure, which in turn reduced the incidence of AKI and achieved effective clinical outcomes with a lower drug burden [21]. Moving forward, adhering to clinical guidelines recommending that TDM be performed within 24 h (or at most 48 h) after vancomycin initiation and that drug regimens be continuously adjusted based on AUC values could further reduce antibiotic use, achieve optimal vancomycin serum concentrations more efficiently, and potentially shorten treatment durations.

The findings of this study highlight that AUC-based vancomycin dosing significantly reduces the incidence of AKI compared to trough-based dosing. It also lowers direct drug costs and decreases additional healthcare expenses associated with AKI management. These results underscore the importance of adopting AUC-based monitoring in clinical practice to optimize vancomycin therapy, improve patient safety, and enhance cost-effectiveness. Furthermore, we plan to conduct additional research to evaluate further the benefits of AUC-based vancomycin dosing in high-risk populations, including patients with impaired renal function, critically ill patients, and elderly patients. This research will provide more robust evidence to support its clinical implementation.

This study has several limitations. First, this study was designed to evaluate the nephrotoxicity of the AUC-based TDM strategy compared to the trough-based TDM strategy in vancomycin administration; therefore, it could not assess treatment efficacy. Second, as a single-center study, the findings may not be generalizable to all patient populations in Korea. Third, because renal function was assessed based on SCr levels, all patients included in the study had normal renal function prior to vancomycin administration. This may have led to an underestimation of the true AKI incidence. Fourth, the two comparison groups were defined based on the time when the institutional vancomycin dosing protocol transitioned from trough-based to AUC-based monitoring. As a result, it is not possible to completely rule out potential temporal confounding factors. Despite these limitations, the study has important strengths. We adjusted for potential confounders such as patient weight, gender, age, renal function, and concomitant nephrotoxic medications using propensity score matching to enhance the robustness of the findings. Additionally, the study period, spanning from 2021 to 2023, was relatively short, reducing the risk of bias due to evolving clinical practices. To our knowledge, this study is the first in Korea to report clinical outcomes after implementing AUC-based vancomycin dosing in a real-world clinical setting.

## 5. Conclusions

AUC-based vancomycin dosing reduced AKI incidence and total drug exposure in adult patients with normal renal function compared to traditional trough-based dosing. These findings highlight the potential of AUC-guided therapeutic drug monitoring to optimize vancomycin therapy. Future studies are warranted to further investigate the benefits of AUC-based dosing when clinical guidelines are followed, including timely blood sampling and therapeutic drug monitoring. Such studies could provide additional insights into its broader applicability and impact on treatment outcomes.

## Figures and Tables

**Table 1:** Patient Characteristics Before and After Weighting by Propensity Score.

Patient Characteristics	Before Weighted by Propensity Score				After Weighted by Propensity Score			
	Trough-Based TDM Group (n = 163), n (%)	AUC-Based TDM Group (n = 290), n (%)	*p*-Value	Variance Ratio	Trough-Based TDM Group (n = 455), n (%)	AUC-Based TDM Group (n = 452), n (%)	*p*-Value	Variance Ratio
Age (year) ^†^	63.7 ± 16.73	62.7 ± 14.07	0.5503	0.7070	63.1 ± 28.04	63.0 ± 17.42	0.9551	0.7035
Body weight (kg) ^†^	63.3 ± 19.39	63.6 ± 16.63	0.8677	0.7351	64.1 ± 34.16	63.7 ± 20.61	0.8238	0.6289
Sex			0.7293	0.9767			0.9598	0.9970
Men	107 (65.6)	195 (67.2)			303 (66.6)	300 (66.4)		
Women	56 (34.4)	95 (32.8)			152 (33.4)	152 (33.6)		
SCr (mg/dL) ^†^	0.78 ± 0.41	0.77 ± 0.36	0.7780	0.7685	0.77 ± 0.61	0.78 ± 0.49	0.8277	1.1256
BUN (mg/dL) ^†^	19.4 ± 11.33	17.2 ± 8.27	0.0265 *	-	19.1 ± 18.38	17.5 ± 10.65	0.1133	-
CLcr (mL/min) ^†^	74.2 ± 26.42	76.2 ± 25.88	0.5317	-	75.6 ± 43.43	75.4 ± 32.59	0.9548	-
eGFR (mL/min/1.73 m^2^) ^†^	91.5 ± 18.91	94.3 ± 15.21	0.1040	-	92.31 ± 29.04	93.99 ± 19.97	0.3084	-
High-risk medication use	21 (12.9)	17 (5.9)	0.0097 *	0.4917	38 (8.4)	37 (8.3)	0.9511	1.0016
Low-risk medication use	117 (71.78)	190 (65.5)	0.1711	1.1153	307 (67.5)	306 (67.6)	0.9750	0.9947
Indication for vancomycin								
Bloodstream infection	50 (30.7)	94 (32.4)	0.7028	1.0302	139 (30.5)	143 (31.6)	0.7548	1.0323
Pneumonia	23 (14.1)	22 (7.6)	0.0259 *	0.5785	45 (9.9)	44 (9.7)	0.9837	1.0117
Neutropenic fever	12 (7.3)	16 (5.5)	0.4339	0.7643	29 (6.4)	29 (6.4)	0.9515	0.9434
Osteomyelitis	20 (12.3)	69 (23.8)	0.0031 *	1.6844	94 (20.7)	89 (19.7)	0.7180	0.9563
Others	58 (35.6)	89 (30.7)	0.2857	0.9280	148 (32.5)	147 (32.5)	0.9635	1.0008
Comorbidity								
HTN	20 (12.3)	51 (17.6)	0.1352	1.3464	73 (16.1)	71 (15.8)	0.9067	0.9740
DM	24 (14.7)	57 (19.7)	0.1887	1.2577	84 (18.5)	82 (18.1)	0.8860	0.9678

^†^ Data are expressed as mean ± SD (standard deviation). * Denotes statistical significance (*p* < 0.05). TDM, therapeutic drug monitoring; AUC, area under curve; SCr, serum creatinine; BUN, blood urea nitrogen; CLcr, creatinine clearance; eGFR, estimated glomerular filtration rate; HTN, hypertension; DM, diabetes mellitus.

**Table 2:** Incidence of AKI Before and After Weighting by Propensity Score.

	Before Weighted by Propensity Score				After Weighted by Propensity Score			
	Trough-Based TDM Group (n = 163) n (%)	AUC-Based TDM Group (n = 290) n (%)	OR (95% CI)	*p*-Value	Trough-Based TDM Group (n = 455) n (%)	AUC-Based TDM Group (n = 452) n (%)	OR (95% CI)	***p*** **-Value**
no AKI	154 (94.48)	287 (98.97)	0.18 (0.05–0.67)	0.0107 *	432 (94.92)	447 (98.80)	0.23 (0.09–0.59)	0.0021 *
AKI	9 (5.52)	3 (1.03)			23 (5.08)	5 (1.20)		

* Denotes statistical significance (*p* < 0.05). AKI, acute kidney injury; TDM, therapeutic drug monitoring; AUC, area under curve; OR, odds ratio.

**Table 3:** The Results of Secondary Outcomes Before and After Propensity Score Weighting.

	Before Weighted by Propensity Score			After Weighted by Propensity Score		
	Trough-Based TDM Group (n = 163) mean ± SD	AUC-Based TDM Group (n = 290) mean ± SD	*p*-Value	Trough-Based TDM Group (n = 455) mean ± SD	AUC-Based TDM Group (n = 452) mean ± SD	*p*-Value
Total dose of vancomycin administered (mg)	29,092 ± 33,512.8	21,093 ± 25,478.3	0.0086 *	29,530 ± 54,671.7	20,987 ± 33,124.1	0.0047 *
Total duration of vancomycin treatment (days)	13.47 ± 13.54	12.35 ± 14.85	0.4280	13.59 ± 22.91	12.35 ± 19.54	0.3811
The number of modifications to the vancomycin regimen	1.08 ± 1.56	1.07 ± 1.52	0.9427	1.09 ± 2.66	1.06 ± 1.89	0.8695

* Denotes statistical significance (*p* < 0.05). TDM, therapeutic drug monitoring; AUC, area under curve; SD, standard deviation.

## Data Availability

Data are available from the corresponding author upon reasonable request.

## Abbreviations

The following abbreviations are used in this manuscript:

AKI	acute kidney injury
ASHP	American society of health-system pharmacists
AUC	area under the curve
BUN	blood urea nitrogen
CI	confidence intervals
CLcr	creatinine clearance
DM	diabetes mellitus
eGFR	estimated glomerular filtration rate
HTN	hypertension
IDSA	infectious diseases society of America
IPTW	inverse probability of treatment weighting
KDIGO	the kidney disease: improving global outcomes
MIC	minimum inhibitory concentration
MRSA	methicillin-resistant *staphylococcus aureus*
OR	odds ratio
SCr	serum creatinine
SD	standard deviation
SIDP	society of infectious diseases pharmacists
STROBE	strengthening the reporting of observational studies in epidemiology
TDM	therapeutic drug monitoring
